# Intrinsic Cell Stress is Independent of Organization in Engineered Cell Sheets

**DOI:** 10.1007/s13239-016-0283-9

**Published:** 2016-10-24

**Authors:** Inge A.E.W. van Loosdregt, Sylvia Dekker, Patrick W. Alford, Cees W.J. Oomens, Sandra Loerakker, Carlijn V.C. Bouten

**Affiliations:** 1 Department of Biomedical Engineering, Eindhoven University of Technology, PO Box 513, 5600 MB Eindhoven, The Netherlands; 2 Institute for Complex Molecular Systems, Eindhoven University of Technology, Eindhoven, The Netherlands; 3 Department of Biomedical Engineering, University of Minnesota, Minneapolis, MN USA

**Keywords:** Cardiovascular tissue engineering, Mechanotransduction, Cell alignment, Finite element modeling

## Abstract

Understanding cell contractility is of fundamental importance for cardiovascular tissue engineering, due to its major impact on the tissue’s mechanical properties as well as the development of permanent dimensional changes, e.g., by contraction or dilatation of the tissue. Previous attempts to quantify contractile cellular stresses mostly used strongly aligned monolayers of cells, which might not represent the actual organization in engineered cardiovascular tissues such as heart valves. In the present study, therefore, we investigated whether differences in organization affect the magnitude of intrinsic stress generated by individual myofibroblasts, a frequently used cell source for *in vitro* engineered heart valves. Four different monolayer organizations were created via micro-contact printing of fibronectin lines on thin PDMS films, ranging from strongly anisotropic to isotropic. Thin film curvature, cell density, and actin stress fiber distribution were quantified, and subsequently, intrinsic stress and contractility of the monolayers were determined by incorporating these data into sample-specific finite element models. Our data indicate that the intrinsic stress exerted by the monolayers in each group correlates with cell density. Additionally, after normalizing for cell density and accounting for differences in alignment, no consistent differences in intrinsic contractility were found between the different monolayer organizations, suggesting that the intrinsic stress exerted by individual myofibroblasts is independent of the organization. Consequently, this study emphasizes the importance of choosing proper architectural properties for scaffolds in cardiovascular tissue engineering, as these directly affect the stresses in the tissue, which play a crucial role in both the functionality and remodeling of (engineered) cardiovascular tissues.

## Introduction

Many cell types exert contractile stresses onto their surroundings via stress fibers.^8^,^10^,^24^,^52^ The main contractile components of the stress fibers are the actin fibers and myosin motors.^8^,^39^,^52^ The stress fibers can be externally stimulated to exert stress (defined here as active cell stress) but also generate an intrinsic level of stress without any external stimulation (defined here as intrinsic cell stress). Understanding and controlling the degree of cell stress is important for tissue engineering in order to obtain mechanically functioning tissues with a proper matrix organization. In cardiovascular tissue engineering, for example, excessive (intrinsic) cellular stress can lead to tissue contraction, represented by leaflet shortening in case of tissue-engineered heart valves (TEHVs).^11^,^30^,^44^ Conversely, insufficient levels of (intrinsic) cellular stress can cause tissue dilation, resulting in leaflet elongation in TEHVs and aneurysm formation in vascular grafts.^42^ The magnitude and direction of cellular stresses depend on the (mechanical) environment, e.g., on stiffness, applied strain, or architecture. For example, previous studies have demonstrated that stiff substrates induce an increased development of stress fibers and focal adhesions compared to soft substrates, enabling the cells to exert increased contractile stresses onto their surroundings.^8^,^10^,^19^,^29^,^51^ Furthermore, cyclic uniaxial strain causes the cells and stress fibers to orient perpendicular to the strain; a phenomenon known as strain avoidance behavior.^16^,^25^,^36^ This causes the cells to exert stresses in the direction perpendicular to the strain, changing the stress directionality. Another phenomenon that affects cell orientation is contact guidance, where the cells align in the direction of topographical environmental cues.^41^

Numerous studies have been performed to improve our understanding of stress fiber remodeling and cellular stress development, both in 2D and in 3D environments. In 3D, most studies have investigated the compaction of fibroblast-loaded gels as a measure of cell contractility.^4^,^6^,^7^,^27^ In addition, studies have been performed to investigate the influence of external cues, such as stiffness,^29^ cyclic strain,^16^ soluble factors^22^ or combinations of stimuli,^17^,^27^ on the actin organization in relation to compaction. Cellular stress development has also been examined in endogenously produced extracellular matrices.^44^,^46^–^48^ Even though 3D studies are more relevant for tissue engineering, extracting (individual) cell stress is challenging due to the complexity of the environment in which the cells reside. 2D studies with cells cultured on substrates may therefore be more suitable for providing the fundamental insights into stress fiber remodeling and cellular stress development.

Various experimental methods exist for measuring cell contractility in 2D,^28^ ranging from single cell methods like traction force microscopy^39^,^53^ and set-ups using micropost arrays,^19^ to monolayer methods such as cyclic stretching of cell monolayers on flexible substrates^25^ and the thin film method.^1^,^21^ The latter method is a suitable method for quantifying both stress fiber remodeling and stress development with minimal handling of the cells.

The thin film consists of a thin layer of polydimethylsiloxane (PDMS) that is attached to a glass substrate via the temperature sensitive polymer poly-*N*-isopropylacrylamide (pIPAAm).^14^,^20^ The PDMS is subsequently micro-contact printed with lines of extracellular matrix proteins to enhance cell adhesion and guide the cells into a specific direction. Rectangular films are typically cut from the PDMS, which partly release from the glass when the pIPAAM dissolves upon a decrease in cell culture medium temperature.^1^,^14^,^20^ The curvature of the films as a result of the contractile cell layer on top of the PDMS can then be used to quantify the stress exerted by the monolayer of cells. The thin film method is therefore an elegant method for determining the contractile properties of aligned contractile tissues, such as smooth,^2^,^50^,^53^ skeletal^40^ or cardiac^21^,^31^ muscle tissue.

Tissue engineered heart valves and blood vessels can be created using (electrospun) fibrous scaffolds^5^,^34^,^37^,^43^ that allow for cell infiltration because of their high porosity. In this case the initial cell alignment is determined by the scaffold fiber organization via the mechanism of contact guidance. As the scaffold fiber organization is highly variable and never perfectly aligned, the cell organization is also not perfectly aligned, and it may be questioned whether the stress exerted by perfectly aligned monolayers represents the stress distribution inside these engineered tissues. In fact, previous studies with cardiomyocytes have shown that the stress developed by a complete monolayer increases upon increasing cellular anisotropy.^13^,^26^,^32^ However, these studies have not investigated whether this was due to differences in intrinsic contractility of the cells, or rather due to differences in alignment.

We hypothesize that the intrinsic contractility of individual cells is independent of the monolayer organization, which would implicate that the intrinsic stress generated by the complete monolayer will be dictated by the cellular organization only. To investigate this hypothesis, we will focus on myofibroblasts derived from the saphenous vein in this study as this cell type is commonly used for creating tissue engineered cardiovascular tissues.^11^,^33^,^34^,^38^,^49^ We adapted the thin film method developed by Feinberg *et al*.,^14^ Alford *et al*.^1^ and Grosberg *et al*.^21^ to determine the intrinsic stress developed by a monolayer of myofibroblasts with various degrees of alignment. The differences in alignment were obtained by seeding the cells onto micro-contact printed fibronectin patterns with different orientations with respect to the long axis of the films. The thin film method was combined with live imaging to determine the curvature and cell density of each individual film. Separate samples were used to stain the cell nuclei, F-actin and phosphorylated myosin light chain, to determine nuclear and stress fiber organization and provide insight into intrinsic stress fiber contraction potential. These experimental results were then combined with sample-specific finite element modeling to determine the intrinsic stress exerted in the film direction by the complete monolayer and the normalized intrinsic cellular contractility.

## Materials and Methods

### Construct Fabrication

Thin film constructs were fabricated as previously described.^1^,^2^,^20^ In brief, a layer of poly-*N*-isopropylacrylamide (pIPAAm; Sigma, Zwijndrecht, The Netherlands) and a layer of polydimethylsiloxane (PDMS; Sylgard 184; Dow Corning, Auburn, MI) were spin coated on a 25 mm diameter glass cover slip and cured overnight at 65 °C. 2.5% of blue silicon dye (Silc-Pig; Smooth-On, Macungie, PA) was added to the PDMS in order to visualize the films and different rotation speeds were used to create PDMS films with different thicknesses in order to account for the difference in film curvature between the different monolayer organizations. In addition, PDMS was spin coated on copper coated glass cover slips used for thickness measurements with an optical profilometer (Plµ 2300; Sensofar, Terrassa, Spain). In order to determine the elastic modulus of the dyed PDMS, rectangular bars were uniaxially strained with a tensile tester (Z010; Zwick/Roell, Venlo, The Netherlands).

### Micro-Contact Printing

**Figure 1:**
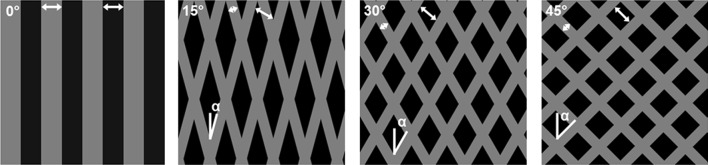
Schematic overview of the micro-contact printing layout of the four different fibronectin patterns. The fibronectin lines are depicted in grey, the spacing in black and the angle α is depicted in the top left corner. The short arrows represent 5 *µ*m and the long arrows represent 10 *µ*m.

PDMS stamps were fabricated using standard photolithography techniques.^23^ The stamps contain features of either 10 *µ*m wide lines with 10 *µ*m spacing in between or a fishnet pattern with 5 *µ*m wide lines with 10 *µ*m spacing at an angle of ±15°, 30° or 45° with respect to the 0° axis (Fig. 1). PDMS stamps were incubated with 50 mg/mL rhodamine fibronectin (Cytoskeleton, Denver, CO) in PBS for one hour, after which they were dried using compressed air. The thin film constructs were treated with UV-ozone (PDS UV-ozone cleaner; Novascan, Ames, IA) for 8 minutes just before transfer of the fibronectin onto the constructs. The stamps were positioned in such a way that the 0° axis of the stamp coincided with the length direction of the to be cut films. After 10 minutes of conformal contact, the constructs were rinsed three times with PBS and stored in PBS at 4 °C until use.^1^,^23^

### Cell Seeding and Culture

Human myofibroblasts were harvested from the vena saphena magna obtained from patients according to Dutch guidelines of secondary used material and were seeded at passage 7 onto the thin film constructs at a seeding density of 8400 cells/cm^2^. After seeding, the HVSCs were cultured at 37 °C and 5% CO_2_ for 2 days in growth medium consisting of Advanced DMEM (Invitrogen, Breda, The Netherlands) supplemented with 10% Fetal Bovine Serum (Greiner Bio-One), 1% GlutaMax (Invitrogen) and 1% penicillin/streptomycin (Lonza, Basel, Switzerland).

### Cell Orientation Analysis

After culture, half of the samples were fixated in 3.7% formaldehyde (Merck, Schiphol-Rijk, The Netherlands) for 15 minutes, permeabilized with 0.5% Triton-X 100 (Merck) for 5 minutes and subsequently incubated for 90 minutes with 2% BSA (Roche, Almere, The Netherlands)—1% horse serum (Sigma) in TBS supplemented with 0.05% Tween (Merck) to block non-specific binding. Mouse anti-phosphomyosin light chain IIA (Cell Signaling, Danvers, MA) was used to label phosphorylated myosin IIA overnight before addition of biotin labeled horse-anti-mouse secondary antibody (Vector, Burlingame, CA) for 90 minutes. Thereafter, the samples were incubated with streptavidin-Alexa 647 (Invitrogen) and phalloidin-Atto 488 (Sigma) for 90 minutes. Before mounting with mowiol (Sigma), the samples were incubated with DAPI (Sigma) for 10 minutes. The mounted samples were visualized using both fluorescent (Axiovert 200 M; Zeiss, Sliedrecht, The Netherlands) and confocal microscopy (LSM 510 Meta; Zeiss). Fluorescent microscopy images at 20 times magnification were analyzed using custom Matlab (MathWorks, Natick, MA) scripts to determine the actin fiber and nuclear orientation with respect to the fibronectin orientation. The actin fiber orientation was determined as described previously.^16^–^18^ For each image a histogram containing the fiber percentage per angle was obtained. The actin stress fiber distribution was subsequently quantified by fitting the following curve to each histogram:

1 $$\varphi_{sf}^{i} = A\left( {c + \exp \left( {\frac{{ - \left( {\gamma - \mu } \right)^{2} }}{{2\sigma^{2} }}} \right)} \right)$$

with the main fiber direction (*μ*) in the 0° direction, *γ* the fiber angle and *A* a scaling factor. The offset (*c*) and dispersity (*σ*) were fit and used as parameters in the finite element model described below (Table 1).

**Table 1:** Overview of specific and common parameters used in the finite element model.

Fibronectin angle	*t*_pdms_ (*µ*m)	*σ* (°)	*c* (–)
0°	8.2	13	0.065
15°	8.2	18	0.086
30°	7.1	23	0.138
45°	6.8	54	2.091

Common parameters	
*t*_cell_ (*µ*m)	3.2
*E*_cell_ (kPa)	0.7
*ν*_cell_ (–)	0.3
*E*_pdms_ (MPa)	1.52/1.91
*ν*_pdms_ (–)	0.49

The nuclear orientation was quantified by thresholding the DAPI image and fitting an ellipse through each nucleus after which the angle of the major axis of the ellipse was determined. As for the actin fibers, histograms were constructed containing the nuclear orientation percentage per angle. The nuclear aspect ratio was calculated by dividing the length of the major axis by the length of the minor axis. Phosphorylated myosin light chain was visualized together with actin at 63 times magnification, to investigate potential co-localization of the two major stress fiber components. The monolayer thickness was determined by analyzing the z-stacks obtained at 40 times magnification with confocal microscopy,^2^ and used as a parameter in the finite element model (Table 1).

### Intrinsic Cell Stress Assay

After culture, the other half of the samples was stained with Hoechst (10 *μ*g/ml; Invitrogen) for 15 minutes, subsequently rinsed 3 times with PBS and growth medium was added to the samples until further use. The stained nuclei were used to determine the nuclear aspect ratio and orientation with respect to the fibronectin lines on each individual film (as described above). In addition, the cell density (*d*) was determined by counting the nuclei in these images. After staining the nuclei, the samples were transferred to a Petri dish with preheated growth medium. The long edges of eight rectangular films were cut from the constructs and the excess PDMS was removed. The petri dish containing the sample was then transferred to a confocal microscope (TCS SP5X; Leica, Son, The Netherlands) to perform temperature- and CO_2_-controlled (37 °C, 5% CO_2_) live imaging of the nuclei and fibronectin lines on the films. Hoechst was excited with a femtosecond pulsed laser (Chameleon; Coherent, Santa Clara, CA) at 750 nm and a laser power of 10%. Rhodamine fibronectin was excited with a white light laser (Leica) at 535 nm and a laser power of 14%. 1024 × 1024 pixel images were taken with a scan speed of 400 Hz. We did not see any adverse effects of the imaging procedure on the cells (data not shown). Next, the ends of the rectangular films were cut while the medium was allowed to cool down below 32 °C in order to dissolve the pIPAAm, and enable the HVSCs to deform the PDMS layer. A picture of the initial curvature (0 h) was taken at room temperature using a stereomicroscope (Discovery.V8; Zeiss) after which the thin films were placed back at 37 °C and 5% CO_2_. Another picture was taken after 1 h when the contractile equilibrium was reached.

### Analysis of Intrinsic Cell Stress

The curvature of the films was determined using Matlab by analyzing the projection length of the bent films.^20^ The length and width of each film were obtained from images of the undeformed films. Thereafter the intrinsic cellular stress was obtained via sample-specific finite element modeling in Abaqus (Dassault Systèmes Simulia Corp., Providence, RI). The cell (*t*_cell_) and PDMS (*t*_pdms_) thickness (Table 1), and the length and width of each film were used as geometrical input for creating a double-layered finite element mesh. Both layers consisted of 200 quadratic brick elements (C3D20), with the bottom layer representing PDMS and the top layer representing the cell monolayer. The PDMS layer was fixed at one of the short edges to represent the experimental setup. The PDMS layer was assigned with compressible Neo-Hookean material properties:

2 $${\varvec{\upsigma}}_{p} = \kappa \frac{\ln J}{J}{\mathbf{I}} + \frac{G}{J}\left( {{\mathbf{B}} - J^{2/3} {\mathbf{I}}} \right)$$

with shear modulus *G* = *E*/2(1 − *ν*), compression modulus *κ* = 2*G*(1 + *ν*)/3(1 − 2*ν*), ***B*** **=** ***F*** **·** ***F*** ^*T*^ and *J* = *det*(***F***), where ***F*** represents the deformation gradient tensor. Parameter values are indicated in Table 1.

The cell layer of the model was a fiber-reinforced layer with an active, fibrous, component (***σ***_*ca*_) representing the stress fibers, and a passive, compressible Neo-Hookean, component (***σ***_*cp*_) representing the other cellular components. ***σ***_*cp*_ was calculated by assuming Neo-Hookean material behavior (Eq. (2); Table 1), while ***σ***_*ca*_ is determined from the stress exerted by the stress fibers in a range of different directions:

3 $${\varvec{\upsigma}}_{ca} = \mathop \sum \limits_{i = 1}^{N} \varphi_{sf}^{i} \sigma_{\hbox{max} } \overset{\lower0.5em\hbox{$\smash{\scriptscriptstyle\rightharpoonup}$}} {e}_{sf}^{i} \overset{\lower0.5em\hbox{$\smash{\scriptscriptstyle\rightharpoonup}$}} {e}_{sf}^{i}$$

where $$\overset{\lower0.5em\hbox{$\smash{\scriptscriptstyle\rightharpoonup}$}} {e}_{sf}^{i}$$ is the stress fiber direction in the deformed configuration, *σ*_max_ is a measure for intrinsic cell contractility, and $$\overset{\lower0.5em\hbox{$\smash{\scriptscriptstyle\rightharpoonup}$}}{\varphi}_{sf}^{i}$$ the actin stress fiber volume fraction for each direction as obtained from the fluorescent images. *σ*_max_ was iteratively increased until the curvature of the finite element models matched the experimentally obtained curvature. The total intrinsic stress in the cell layer of the model was obtained by adding the passive and active stress components: ***σ***_*c*_ = ***σ***_*cp*_ + ***σ***_*ca*_. The magnitude of the intrinsic stress component in the long axis direction of the deformed film ($$\overset{\lower0.5em\hbox{$\smash{\scriptscriptstyle\rightharpoonup}$}} {e}_{la}$$) in the cell layer (*σ*_*f*_) was determined using:

4 $$\sigma_{f} = {\varvec{\upsigma}}_{c} \cdot \overset{\lower0.5em\hbox{$\smash{\scriptscriptstyle\rightharpoonup}$}} {e}_{la} \cdot \overset{\lower0.5em\hbox{$\smash{\scriptscriptstyle\rightharpoonup}$}} {e}_{la}.$$

In order to compare intrinsic cell contractility between samples, we normalized *σ*_max_ for the cell density

5 $$\sigma_{\text{norm}} = \frac{{\sigma_{ \hbox{max} } }}{d}.$$

### Statistical Analysis

Quantitative data were analyzed with SPSS Statistics 22 (IBM, Amsterdam, The Netherlands) and were considered significant at *p* < 0.05. Differences in nuclear aspect ratio were analyzed using a one-way ANOVA with a Bonferroni *post hoc* test. Spearman’s correlation coefficient (*ρ*) was determined to investigate correlations between cell density and either curvature, *σ*_*f*_ or *σ*_max_. Differences in *σ*_norm_ between the four different alignment groups were analyzed using a non-parametric Kruskal–Wallis test with pairwise Wilcoxon rank sum tests with corrected levels as *post hoc* analysis.

## Results

### Monolayer Organization is Determined by the Fibronectin Pattern

**Figure 2:**
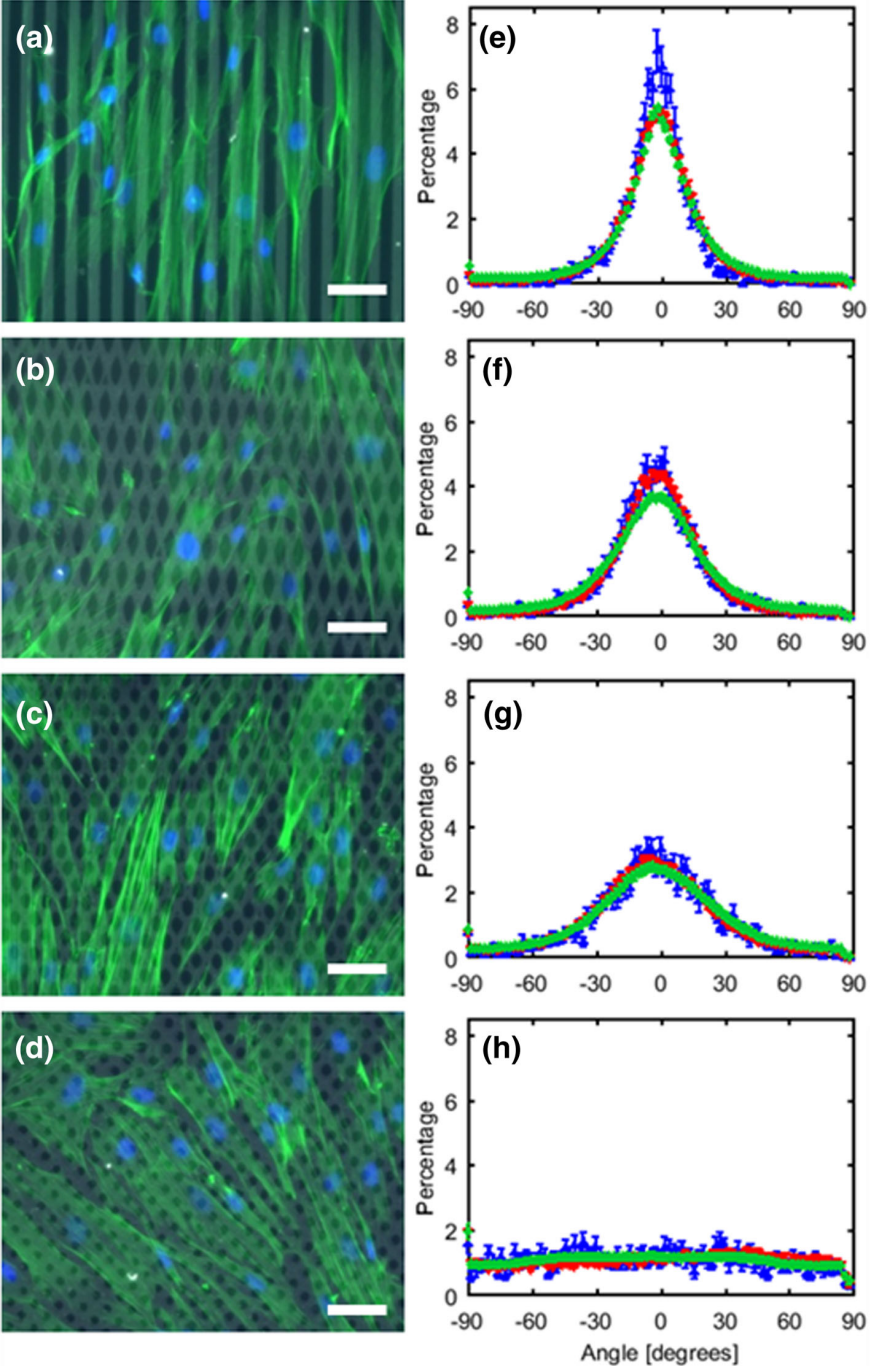
Actin (green) and nuclei (blue) of myofibroblasts cultured on fibronectin lines (grey) with four different orientation angles (a–d; scale bar is 50 *µ*m) and corresponding histograms of the actin and nuclear orientation (e–h; mean ± standard error of mean). The green markers represent the actin fibers (40 images), blue markers represent the nuclei of the stained samples (40 images) and the red markers represent the nuclei of the samples used for stress measurements (26–32 films).

**Figure 3:**
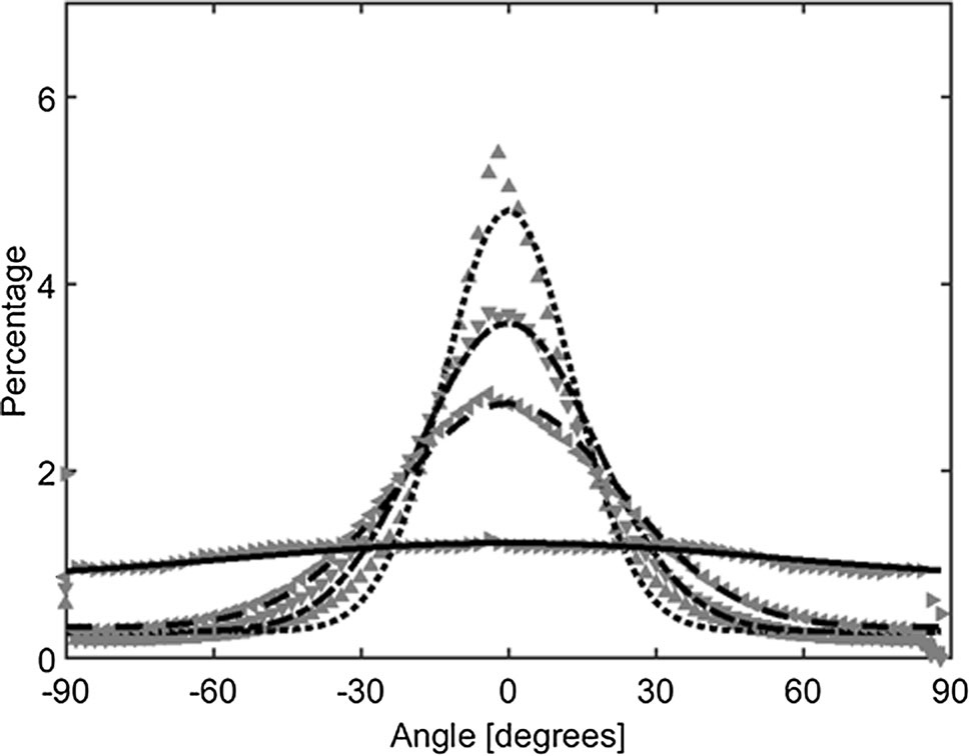
Mean actin orientation (grey triangles) with corresponding fit (black lines) for 0° (upward triangles; dotted line), 15° (downward triangles; dash dot line), 30° (left pointing triangles; dashed line), and 45° (right pointing triangles; solid line).

The nuclear and actin orientation of the cells on substrates with different orientations of fibronectin lines is shown in Fig. 2. For all groups, the orientation of the actin fibers, the nuclei of the samples used for staining, and the nuclei of the samples used for the stress measurements coincided. This indicates that both the actin orientation and the nuclear orientation are a good measure for overall cellular orientation. The cells of the 0° group were primarily aligned in the direction of the fibronectin lines, as demonstrated by the high peak in the orientation histograms at 0° for both the actin fibers and nuclei (Figs. 2a and 2e). Upon increasing the angle between the fibronectin lines, the peak at 0° flattened out until a completely random orientation was reached in the 45° group (Fig. 2d and 2h). The mean actin fiber distribution of each group was successfully fit using Eq. (2) (Fig. 3). The obtained parameters are shown in Table 1, and served as input for the computational model.

The nuclear aspect ratio was determined to be 1.74 ± 0.09, 1.65 ± 0.05, 1.61 ± 0.05, and 1.60 ± 0.06 for the 0°, 15°, 30°, and 45° group, respectively. This indicates that the nuclei were all elliptical and significantly different from each other (*p* < 0.002), except for the aspect ratio of the nuclei on the 30° and 45° fibronectin lines.

### Curvature Increases with Increasing Cell Density

**Figure 4:**
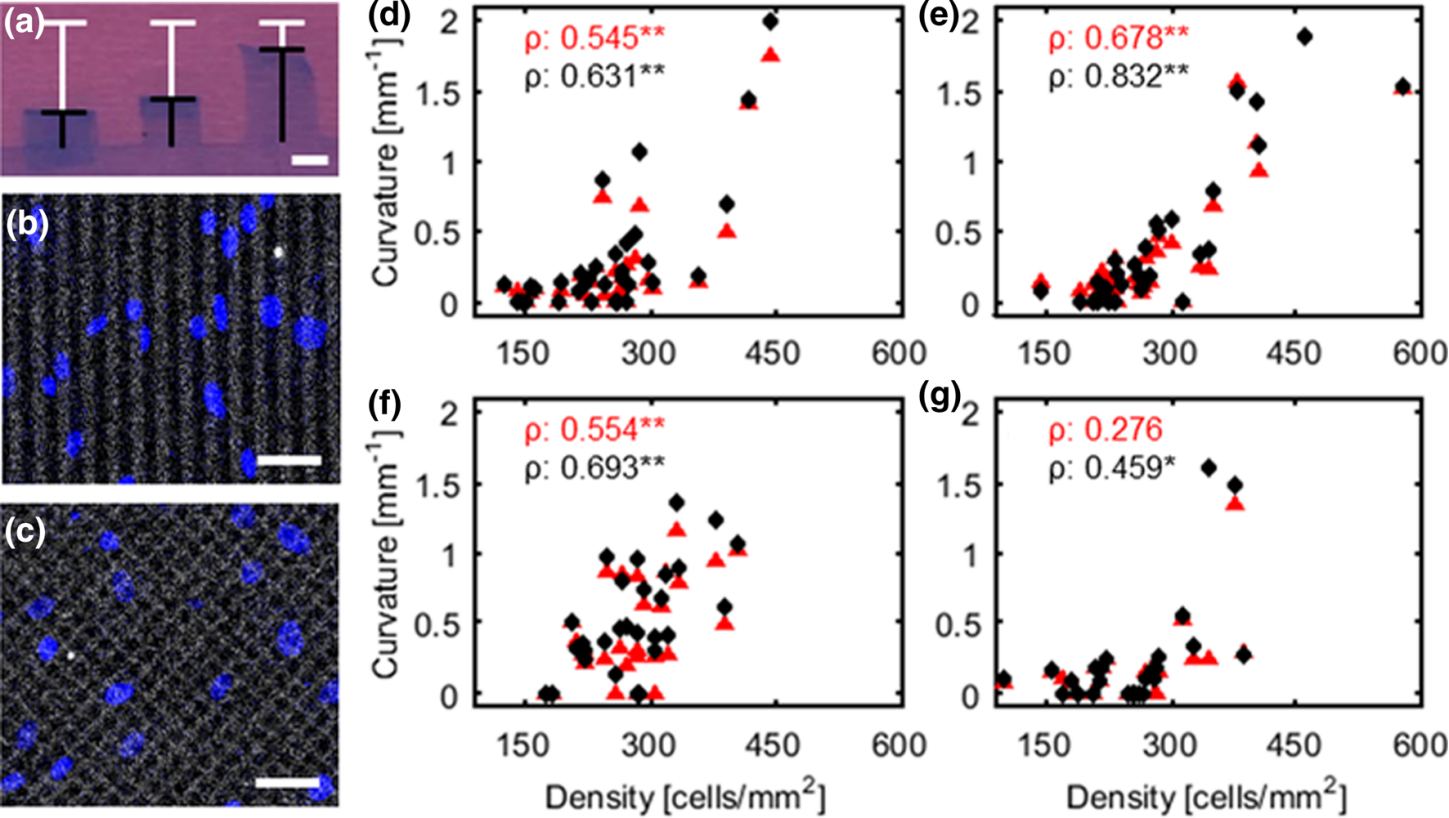
Representative top view image of bent films with 30° fibronectin lines at equilibrium (1 h), the black bars represent the projection length and the white bars the initial length; scale bar is 1 mm (a). Examples of confocal images of nuclei (blue) on fibronectin lines (grey) at 0° (b) and ±45° (c); scale bar is 50 *µ*m. Density-curvature plots of the films at 0 h (red triangles) and 1 h (black diamonds) for the 0° (d), 15° (e), 30° (f), and 45° (g) groups. Spearman’s correlation coefficient is depicted in the top left corner for each density-curvature plot (n = 26–32). *p < 0.05, **p < 0.01.

For all groups, 32 films were manufactured to perform curvature measurements on. In case of discontinuities in the fibronectin pattern, the film was not included in the analysis. This resulted in analysis of respectively 32, 31, 29, and 26 films for the 0°, 15°, 30°, and 45° groups. Considerable differences in curvature between films within the same group were present (Fig. 4a), due to local variations in cell density. Therefore, the number of nuclei on each film was quantified via a Hoechst staining (Figs. 4b, 4c) in order to determine the correlation between cell density and film curvature for each group at both time points. For all groups, positive correlations between cell density and curvature were found, except for the 45° group at 0 h (Fig. 4d–4g). In addition, a minimum cell density was required for the cells to be able to bend the film, which approximately equaled 150–200 cells/mm^2^.

### Normalized Intrinsic Contractility Seems Independent of Monolayer Alignment

When film curvature was absent, the intrinsic stress exerted by the monolayer was lower than the measurement limit of this method. In that case, finite element simulations were omitted. Simulations were performed for the remaining 151 films (both time points included). 26 simulations failed before reaching the experimentally observed curvature due to convergence issues, and were excluded from further analysis. Due to the lack of remaining samples with a cell density above 300 cells/mm^2^ in the 0° group, no clear correlation was found between the cell density and the intrinsic stress in the direction of the film (*σ*_*f*_) for this group. For 15° samples there was a significant correlation between the cell density and *σ*_*f*_. A similar correlation was observed for the 30° samples, albeit with larger dispersity. For the samples with ±45° fibronectin lines a significant correlation was observed at 0 h, however not as strong as for the 15° and 30° samples, probably also due to the lack of samples with a high cell density. As the intrinsic stress in all directions is taken into account in *σ*_max_ (Eq. (3)), its value was higher than *σ*_*f*_, which only includes the intrinsic stress in the direction of the film (Eq. (4)). Naturally, the difference between *σ*_max_ and *σ*_*f*_ increased with increasing fibronectin angle. No significant differences in *σ*_norm_ were found between groups with the exception of the 30° group being significantly higher compared to the 0° group at 0 and 1 h. The median *σ*_norm_ ranged between 3.43 and 6.76 Pa at 0 h and between 4.80 and 8.76 Pa at 1 h (Fig. 6).

### Stress Fiber Organization is Similar in All Groups

Stainings for actin and phosphorylated myosin light chain, the major stress fiber components, are shown in Fig. 7. In all groups, actin fibers were abundantly present and oriented along the longitudinal direction of the cells. Phosphorylated myosin light chain was observed to co-localize with the actin fibers, confirming the ability of the stress fibers to contract.

## Discussion

Understanding cell contractility is of fundamental importance for cardiovascular tissue engineering, due to its major impact on the tissue’s mechanical properties as well as the development of permanent dimensional changes, e.g., by contraction or dilatation of the tissue. In previous attempts to quantify the contractile cellular stresses by means of the thin film method,^2^,^21^,^31^,^40^,^50^,^53^ mostly strongly aligned monolayers of cells were used, which might not represent the actual cellular organization in engineered cardiovascular tissues. In the present study, we investigated whether differences in alignment would affect the magnitude of the intrinsic stress generated by individual myofibroblasts. We hypothesized that the intrinsic contractile stress exerted by the myofibroblasts is independent of the monolayer organization, as a result of which the total intrinsic stress exerted by the monolayer should be dictated by the actual cell alignment. To test our hypothesis, patterns of fibronectin lines were micro-contact printed on thin film constructs in order to create monolayers with varying degrees of cell alignment. The intrinsic stress exerted by each monolayer in the direction of the film was determined from the curvature of the thin films, and was found to correlate positively with the cell density. Importantly, after accounting for differences in cell alignment and normalizing for cell density, no consistent differences in intrinsic cellular contractility were found between the different monolayer organizations, suggesting that the intrinsic stress exerted by monolayers of myofibroblasts can indeed be predicted from the cellular organization. These findings are supported by the similarity in staining for stress fiber organization observed in the different groups.

Using a simple and controlled method consisting of micro-contact printing different fibronectin patterns, cell sheets with organizations ranging from highly aligned to completely random were successfully created. As the actin fiber orientation and the nuclear orientation were overlapping (Fig. 2e–2h), both appeared to be good indicators of cell orientation and organization. Since previous research has shown that the nuclear aspect ratio is correlated with the cellular aspect ratio,^2^,^50^,^53^ we used this measure as an indicator of cellular shape. In all conditions, the aspect ratio was larger than 1.60 indicating the presence of elliptical nuclear shapes and thus elongated cells (Fig. 2a–d). The nuclear aspect ratio increased upon increasing cellular alignment, suggesting that the cells adopted a more elongated shape for higher degrees of cellular alignment.

**Figure 5:**
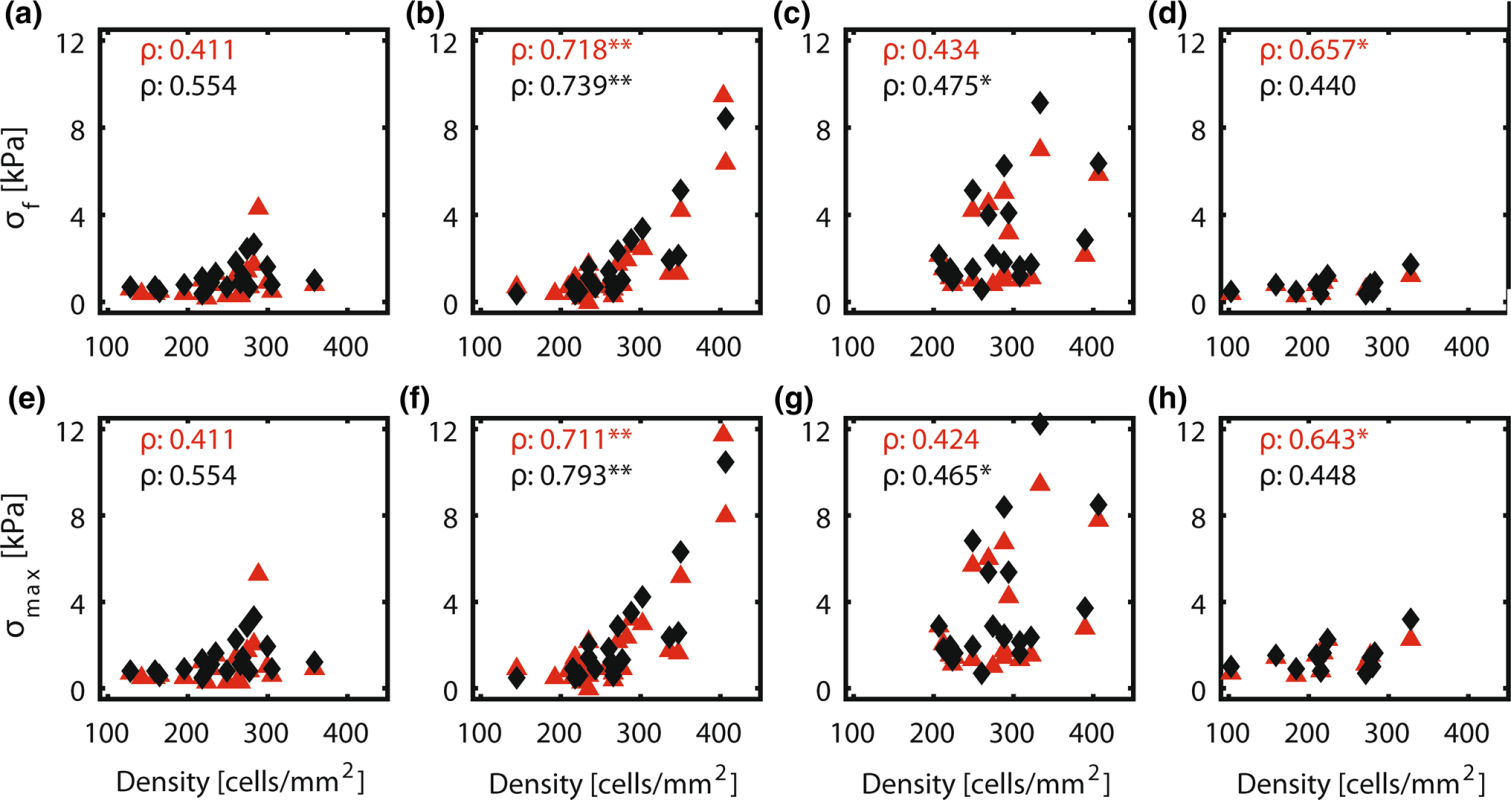
Density–stress plots for the stress in the length direction of the film (σ_f_; a–d) and for the maximum stress fiber stress (σ_max_; e–h) for the 0° (a, e), 15° (b, f), 30° (c, g), and 45° (d, h) group at 0 h (red triangles) and 1 h (black diamonds). Spearman’s correlation coefficient is depicted in the top left corner of each density-stress plot (n = 13-25). *p < 0.05, **p < 0.01.

The minimum cell density that was required to induce significant curvature of the films was 150–200 cells/mm^2^, regardless of the cellular organization (Fig. 4). In a study that investigated collagen gel compaction by osteoblasts, a comparable threshold value of 100 cells/mm^2^ was found.^15^ When the threshold density was exceeded, both the intrinsic stress in the direction of the film (*σ*_*f*_) and the measure for intrinsic cell contractility (*σ*_max_) correlated with cell density and increased over time (Fig. 5). These correlations were less strong in the 0° and 45° group, probably due to the low number of samples with a high cell density (> 300 cells/mm^2^). Few studies have been published on the effect of cell density on contraction using gel compaction assays without a predefined cellular organization.^9^,^12^,^15^,^35^ Similar to the results obtained in our study, they found that the initial compaction is higher in high-density gels compared to low-density gels. Moreover, the high-density gels also reached the maximum compaction at a faster rate compared to the low-density gels, although the actual maximum was the same for both types of gels. It is however unclear if the maximum degree of compaction in these gels is caused by direct cellular contractility only.

**Figure 6:**
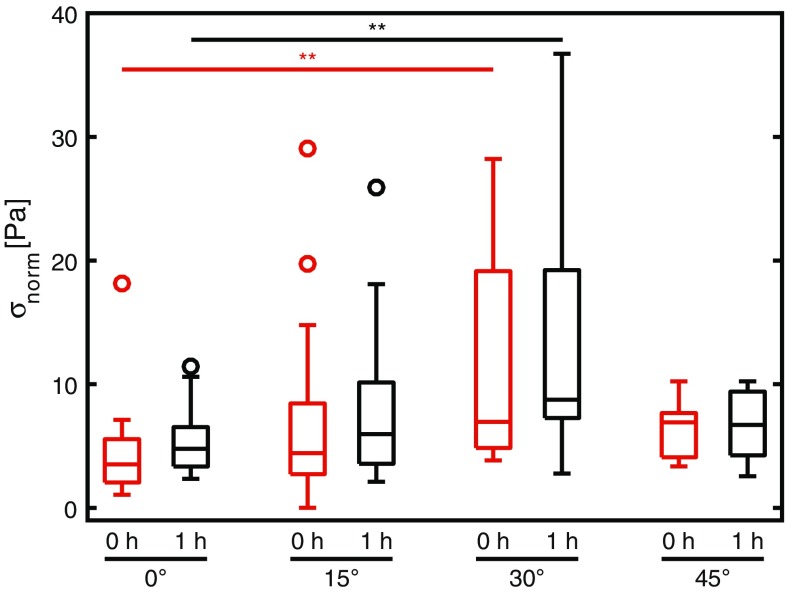
σ_max_ normalized for cell density (σ_norm_) at 0 h (red) and 1 h (black),* n* = 13–25. **p < 0.01.

**Figure 7:**
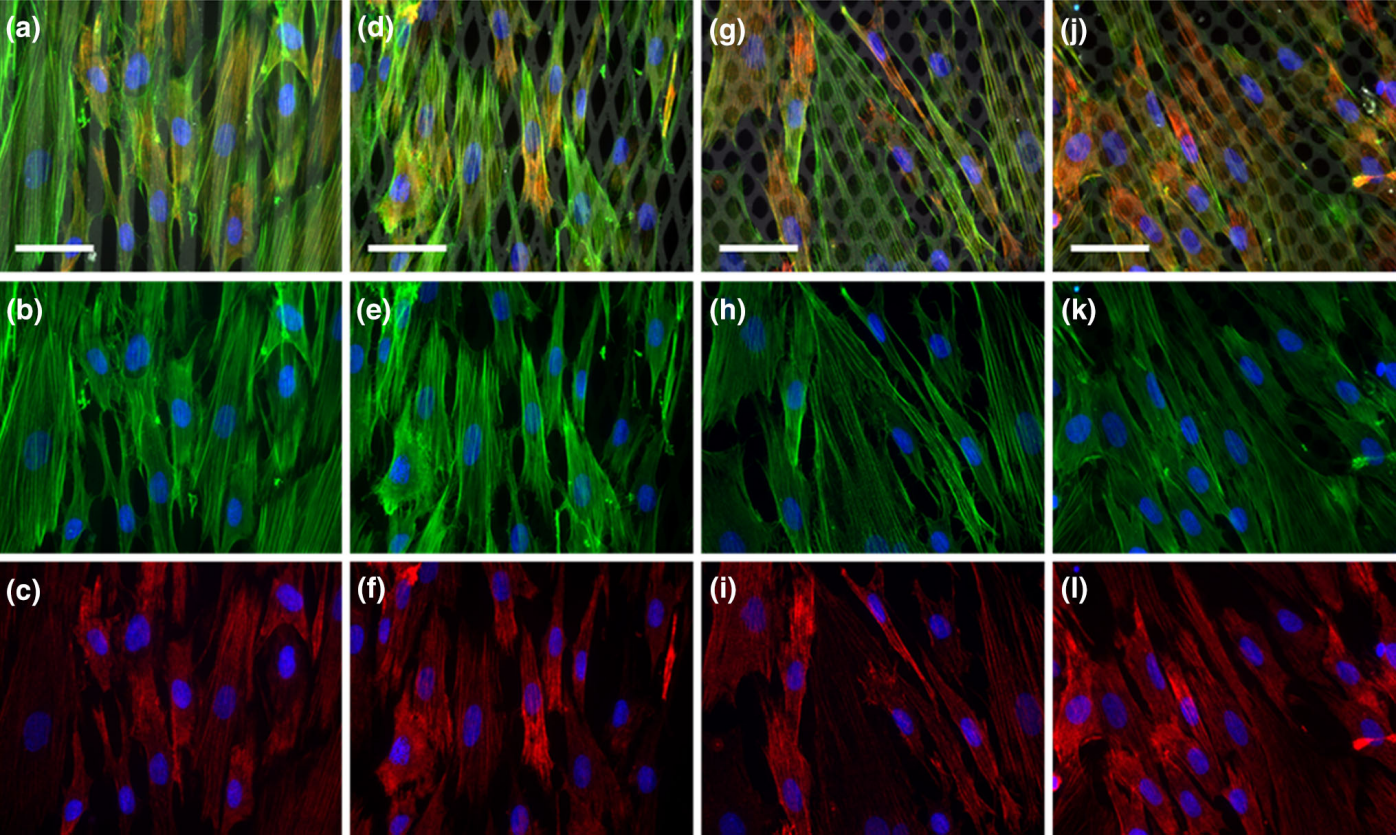
Representative fluorescent microscopy images of actin (green), phosphorylated myosin light chain (red), fibronectin (grey), and nuclei (blue). The angle of the fibronectin lines is 0° in (a–c), 15° in (d–f), 30° in (g–i), and 45° in (j–l). Merged images are shown on the top row (a, d, g, j), actin and nuclei are shown on the middle row (b, e, h, k), and phosphorylated myosin light chain and nuclei are shown on the bottom row (c, f, i, l). Scale bar is 50 *μ*m.

When correcting the intrinsic stress exerted by the monolayer for differences in cell density and cellular organization, significant differences in intrinsic cell contractility (*σ*_norm_; Fig. 6) were only found between the 0° and 30° degree groups, which may be explained by the combined effects of the high spread in *σ*_norm_ at the 30° group, the lack of samples with a high cell density in the 0° group, and the lack of low cell density samples in the 30° group. Taken together, no consistent significant differences in the normalized stress were observed between the different groups (with the median *σ*_norm_ ranging from 3.43 to 8.76 Pa). Therefore, our data suggest that the intrinsic stress exerted by individual myofibroblasts is independent of the monolayer organization. The actin and phosphorylated myosin light chain staining support this finding as no differences in the stress fiber organization were found between groups (Fig. 7).

Previous studies that have investigated the relationship between cell organization and stress development have mainly focused on myocardial tissues.^13^,^26^,^32^,^40^,^45^ Most of these studies observed that the stress developed by the complete tissue is higher when the cells are aligned compared to a random cell organization. In addition to that, similar to this study, Knight *et al*.^26^ recently investigated multiple degrees of anisotropy, demonstrating a doubling of the stress in the film direction of anisotropic myocardial tissue compared to isotropic tissue, with a gradual decrease in global stress with decreasing anisotropy. However, as these stresses were not corrected for differences in alignment and cell density, it remains unclear whether the stress generated by individual cardiomyocytes depends on the local or global cell alignment. This uncertainty is even more emphasized by the fact that two studies that have normalized the globally found cardiac tissue stress are contradictory to each other, where Feinberg *et al*.^13^ concluded that the force generated by individual sarcomeres increases upon increasing alignment, and Van Spreeuwel *et al*.^45^ found that cardiomyocytes in both anisotropic and isotropic tissues exert similar amounts of force.

A limitation of the current study is the presence of a spatial variability in PDMS thickness between samples that were manufactured with the same settings. As the stress that is necessary to bend the thin film is strongly dependent on the magnitude of the thickness,^14^ this variability may have induced some uncertainty in the calculated stresses. Furthermore, it resulted in a decrease in sample size of *σ*_*f*_, *σ*_max_, and *σ*_norm_ compared to the curvature data, due to the fact that in case of 0 mm^−1^ curvature the stresses could not be quantified as a result of low cell densities in combination with relatively high local PDMS thickness. In addition, by normalizing the calculated intrinsic stress for cell density, we assumed that all cells were exerting their intrinsic stress solely onto the PDMS layer, without pulling on their neighboring cells via cell–cell contacts. Future studies should point out whether this assumption is completely valid.

In summary, the results of our study suggest that the individual intrinsic contractility of myofibroblasts is independent of monolayer architecture, implying that the architecture itself dictates the total intrinsic stress distribution in the tissue. With regard to cardiovascular tissue engineering, the initial organization of engineered tissues is often imposed via the presence of fibrous scaffolds.^5^,^34^,^37^,^43^ These scaffolds are essential in delivering the correct material properties that will induce physiological tissue deformations.^3^,^30^ As the scaffold architecture directly determines the direction in which individual cells exert stress and the magnitude of this stress is not affected by the architecture, the total stress distribution in the tissue, generated by all cells in that tissue, is completely determined by the scaffold architecture. Hence, the fact that the intrinsic stress generated by individual cells remains the same implies that the total intrinsic stress distribution can directly be altered by changing the architecture of the scaffold. This plays a crucial role in both the functionality and remodeling of (engineered) cardiovascular tissues.
